# Salinomycin Promotes Anoikis and Decreases the CD44^+^/CD24^-^ Stem-Like Population via Inhibition of STAT3 Activation in MDA-MB-231 Cells

**DOI:** 10.1371/journal.pone.0141919

**Published:** 2015-11-03

**Authors:** Hyunsook An, Ji Young Kim, Eunhye Oh, Nahyun Lee, Youngkwan Cho, Jae Hong Seo

**Affiliations:** 1 Division of Medical Oncology, Department of Internal Medicine, Korea University College of Medicine, Korea University, Seoul 152–703, Republic of Korea; 2 Brain Korea 21 Program for Biomedicine Science, Korea University College of Medicine, Korea University, Seoul 152–703, Republic of Korea; Stony Brook University, UNITED STATES

## Abstract

Triple-negative breast cancer (TNBC) is an aggressive tumor subtype with an enriched CD44^+^/CD24^-^ stem-like population. Salinomycin is an antibiotic that has been shown to target cancer stem cells (CSC); however, the mechanisms of action involved have not been well characterized. The objective of the present study was to investigate the effect of salinomycin on cell death, migration, and invasion, as well as CSC-like properties in MDA-MB-231 breast cancer cells. Salinomycin significantly induced anoikis-sensitivity, accompanied by caspase-3 and caspase-8 activation and PARP cleavage, during anchorage-independent growth. Salinomycin treatment also caused a marked suppression of cell migration and invasion with concomitant downregulation of *MMP-9* and *MMP-2* mRNA levels. Notably, salinomycin inhibited the formation of mammospheres and effectively reduced the CD44^+^/CD24^-^ stem-like population during anchorage-independent growth. These observations were associated with the inhibition of STAT3 phosphorylation (Tyr705). Furthermore, interleukin-6 (IL-6)-induced STAT3 activation was strongly suppressed by salinomycin challenge. These findings support the notion that salinomycin may be potentially efficacious for targeting breast cancer stem-like cells through the inhibition of STAT3 activation.

## Introduction

Triple-negative breast cancers (TNBC) is a malignant subtype that fails to express the estrogen receptor (ER), progesterone receptor (PR), and human epidermal growth factor receptor 2 (HER2), representing 10–20% of all breast cancer cases [1,2]. Accumulating evidence has demonstrated that TNBCs harboring a high percentage of CD44^+^/CD24^-^ cells have a more aggressive phenotype [3–5].

Cancer stem cells (CSC) represent a subpopulation of cancer cells with enhanced properties of self-renewal and differentiation. The presence of CSCs is associated with resistance to treatment and a higher risk of tumor recurrence and cell spreading [6–8]. Because the targeting of CSCs has been viewed as an important strategy for long-lasting treatment, a number of studies have focused on the identification of CSC markers such as CD44, CD24, epithelial cell surface antigen (ESA), and aldehyde dehydrogenase 1 (ALDH1) in breast cancer. It has been demonstrated that a small number of breast cancer cells with the CD44^+^/CD24^-^ phenotype can form tumors after orthotopic injection into the mammary fat pad in immunocompromised mice [9–11]. Of particular note, CSCs are resistant to anoikis, allowing them to replicate under anchorage-independent conditions [12,13].

Anoikis, a specific form of cell death induced by cell detachment from the extracellular matrix (ECM), plays a critical role in inhibiting cell dissemination to distant organs. The suppression of anoikis can therefore facilitate cell growth at inappropriate locations, thereby contributing to metastasis. Anoikis resistance is promoted by various factors including signal transducer and activator of transcription 3 (STAT3), phosphatidylinositol 3-kinase (PI3K)/Akt, focal adhesion kinase (FAK), and Src family tyrosine kinases [14–17]. Notably, STAT3 is an important determinant in CSC fate decision as well as anoikis resistance [3,18–20].

STAT3 belongs to the STAT family of proteins and is involved in cell proliferation and survival via the expression of cyclin D1 and survivin. Clinical studies have revealed that levels of phospho-STAT3 (Tyr705) are elevated in patients with stage III invasive breast cancer, which is closely correlated with poor responses to neoadjuvant therapy [21–23]. STAT3 activation leads to the inhibition of apoptosis and tumor progression in TNBC, and its dysfunction induces cell death *in vitro* and *in vivo*, implicating STAT3 as an attractive therapeutic target [24–26].

Salinomycin, an anticoccidial drug for poultry, was identified by high-throughput screening of 16,000 compounds from chemical libraries for activity against human breast CSCs. The compound was found to inhibit breast CSCs more effectively than conventional anticancer drugs such as paclitaxel [27]. Treatment with salinomycin induced cell death in various solid tumors, associated with the generation of ROS, suppression of cell motility, and induction of autophagy [28–30]. However, the cellular mechanisms responsible for the induction of apoptosis and the regulation of CSC properties in TNBC remain to be elucidated.

The objective of the present study was to examine the effect of salinomycin on cell death, migration, and invasion, and the CSC-like population in MDA-MB-231 breast cancer cells.

## Materials and Methods

### Reagents and antibodies

Salinomycin, S3I-201, interleukin-6 (IL-6), paraformaldehyde (PFA), dimethyl sulfoxide (DMSO), Triton X-100, and phosphate buffered saline (PBS) tablets were obtained from Sigma-Aldrich (St. Louis, MO, USA). LLL12 was purchased from BioVision Inc. (Milpitas, CA, USA). The 2 mM stock solution of salinomycin was dissolved in DMSO and used at final concentrations of 0.5–10 μM. The 20 mM stock solution of S3I-201 and 1 mM stock solution of LLL12 were dissolved in DMSO and used at final concentrations of 50 μM and 1 μM, respectively. An equal volume of DMSO was added to control groups and the final concentration of DMSO was less than 0.5%. Aliquots of all stock solutions were stored at -20°C. Phosphatase inhibitor and protease inhibitor cocktail tablets were purchased from Roche Applied Sciences (Penzberg, Germany). Primary antibodies were used for the following proteins: STAT3, phospho-STAT3 (Tyr705) (Abcam, Cambridge, MA, USA); PARP, cleaved PARP, cleaved caspase-3, cleaved caspase-8 (Cell Signaling Technology, Beverly, CA, USA); cyclin D1, survivin, Bcl-2 (Santa Cruz Biotechnology, Santa Cruz, CA, USA); actin (Sigma-Aldrich). The secondary antibodies used were horseradish peroxidase (HRP)-conjugated anti-rabbit and mouse IgG (Bio-Rad Laboratories, Hercules, CA, USA) and Alexa Fluor-488 goat anti-rabbit and mouse IgG (Invitrogen, Carlsbad, CA, USA).

### Cell culture

The human breast cancer cell lines MCF7, T47D, BT474, MDA-MB-453, SKBR3, Hs578T and MDA-MB-231 (American Type Culture Collection, Manassas, VA, USA), and mouse mammary carcinoma cell line 4T1 were cultured in DMEM or RPMI containing 10% fetal bovine serum (FBS), streptomycin-penicillin (100 U/ml), and Fungizone (0.625 μg/ml). Cells were incubated at 37°C in an atmosphere of 5% CO_2_.

### Cell viability assay

Cell viability was determined using the CellTiter 96* Aqueous One Solution Cell Proliferation Assay [MTS, 3-(4,5-dimethylthiazol-2-yl)-5-(3-carboxymethoxyphenyl)-2-(4-sulfophenyl)-2H-tetrazolium] (Promega, Madison, WI, USA) according to the manufacturer’s instructions. The quantity of formazan product was determined by measuring absorbance at 490 nm using a Spectramax Plus384 microplate analyzer (Molecular Devices, Sunnyvale, CA, USA).

### Western blot analysis

Cells were solubilized in lysis buffer [30 mM NaCl, 0.5% Triton X-100, 50 mM Tris-HCl (pH 7.4)] containing phosphatase and protease inhibitor cocktail tablets. Supernatant was collected after centrifugation (14,000 g, 4°C, 20 min) and protein concentrations were measured with a Bradford protein assay kit (Bio-Rad Laboratories). Equal quantities of protein (30 μg) were subjected to SDS-PAGE and electrotransferred onto a nitrocellulose membrane (GE Healthcare Life Sciences, Buckinghamshire, UK). The membranes were incubated overnight at 4°C with primary antibodies diluted in 5% BSA [JAK2 (1:2,000), phospho-JAK2 (Tyr1007/1008, 1:2,000), STAT3 (1:2,000), phospho-STAT3 (Tyr705, 1:2,000), PARP (1:2,000), cleaved PARP (1:2,000), cleaved caspase-3 (1:1,000), cleaved caspase-8 (1:1,000), survivin (1:2,000), Bcl-2 (1:1,000), cyclin D1 (1:2,000), and actin (1:5,000)], followed by incubation with horseradish peroxidase (HRP)-conjugated anti-rabbit and mouse IgG (1:3,000–1:10,000). Signal intensity was detected using an Enhanced Chemiluminescence Kit (Thermo Scientific Inc., Rockford, IL, USA) and X-ray film (Agfa Healthcare, Mortsel, Belgium) and quantitated using AlphaEaseFC software (Alpha Innotech, San Leandro, CA, USA).

### Sub-G1 assay

Cells were harvested and fixed with 95% ethanol containing 0.5% Tween-20 for 24 h, washed with PBS, and incubated with propidium iodide (PI, 50 μg/ml) and RNase (50 μg/ml) for 30 min. Sub-G1 analysis was conducted by flow cytometry using a Beckman Coulter Expo.

### Annexin V/PI assay and CD44^+^/CD24^-^ staining analysis

Cells were stained using a FITC-conjugated Annexin V apoptosis detection kit (BD Biosciences, Franklin Lakes, NJ, USA) according to the manufacturer’s protocol. For CD44^+^/CD24^-^ staining analysis, cells were incubated for 30 min at 4°C with FITC- and PE-conjugated anti-mouse IgG or FITC-conjugated anti-CD24 and PE-conjugated anti-CD44 antibodies (BD Biosciences). Cells were analyzed by flow cytometry using a Beckman Coulter Expo.

### Mammosphere formation assay

Cells were plated in ultralow attachment dishes (Corning, NY, USA) and cultured in HuMEC basal serum-free medium (Gibco), supplemented with B27 (1:50, Invitrogen, Carlsbad, CA), 20 ng/mL basic fibroblast growth factor (bFGF, Sigma-Aldrich), 20 ng/mL human epidermal growth factor (EGF, Sigma-Aldrich), 4 μg/ml heparin, 1% antibiotic-antimycotic agent, and 15 μg/mL gentamycin. The number and volume of mammospheres were determined under an Olympus IX 71 inverted microscope and photos were acquired with Olympus DP capture software.

### Immunofluorescence confocal microscopy

Cells on 8-well chamber slides (BD Biosciences) were fixed with 4% PFA, washed with PBS, and permeabilized with 0.2% Triton X-100 for 10 min. Cells were incubated with primary antibodies in antibody diluent (Dako, Glostrup, Denmark) overnight at 4°C and then with Alexa Fluor-488 goat anti-rabbit and mouse IgG (Invitrogen) secondary antibodies. Cells were mounted with ProLong Gold Antifade Reagent with DAPI (Life Technologies, Carlsbad, CA, USA). Images were acquired using a Carl Zeiss confocal microscope (Weimar, Germany).

### RT-PCR analysis

Total RNA was extracted using the RNeasy mini kit (Qiagen, Valencia, CA, USA), according to the manufacturer’s instructions. Transcript amplification was achieved by reverse transcriptase-polymerase chain reaction (RT-PCR) using 1 μg/μl total RNA, Molony Murine Leukemia Virus reverse transcriptase (MMLV; Gibco/BRL, Gaithersburg, MD, USA), and oligo-d(T)15 primer (Roche Applied Sciences). PCR amplification was performed using the following primers: MMP-2, forward 5’-TCT CCT GAC ATT GAC CTT GGC-3’, reverse 5’-CAA GGT GCT GGC TGA GTA GAT C-3’; MMP-9, forward 5’-TTG ACA GCG ACA AGA AGT GG-3’, reverse 5’-CCC TCA GTG AAG CGG TAC AT-3’; cyclin D1, forward 5’-ACA CGG ACT ACA GGG GAG TTT-3’, reverse 5’-GGA AGC GGT CCA GGT AGT TC-3’; actin, forward 5’-ACC CAG ATC ATG TTT GAG AC-3’, reverse 5’-GGA GTT GAA GGT AGT TTC GT-3’. The PCR products were separated on 1.2% agarose gels and visualized using a Gel Doc^™^ XR+ System (Bio-Rad Laboratories).

### Matrigel invasion assay

Invasion chambers were coated with Matrigel matrix (BD Biosciences) according to the manufacturer’s recommendations. Cells were treated with salinomycin and then trypsinized, washed, and transferred to the upper chambers in serum-free media. Conditioned media was added to the lower chambers. The chambers were incubated for 48 h at 37°C in a humidified atmosphere of 5% CO_2_. Invaded cells were fixed and stained with Diff-Quik (Sysmex, Kobe, Japan) and counted under a BX51 microscope (Olympus, Tokyo, Japan).

### Wound healing assay

For kinetic migration analysis, cells were seeded to ~90% confluency in 96-well plates (Essen ImageLock, Essen Biosciences, Ann Arbor, MI, USA). Wound areas were made with a 96-pin Wound Maker device and washed with PBS to prevent reattachment of dislodged cells. Cells were treated with salinomycin immediately after wound scratching, and images of the scratched fields were automatically acquired and registered every hour for 24 h with an IncuCyte^™^ ZOOM^®^ Kinetic Imaging System. The relative wound density was analyzed using the IncuCyte^™^ Scratch Wound Cell Migration Software Module.

### Statistical analysis

All data were analyzed using GraphPad Prism 5.0 statistical software (San Diego, CA, USA). The results are presented as mean ± SEM of at least three independent experiments. Data were analyzed by Student’s t test and one- or two-way ANOVA, as appropriate. A two-way ANOVA was used to assess the effects and interactions of two variables and multiple comparisons were performed using the Bonferroni *post hoc* test. Statistical significance was defined at *p* < 0.05 (*).

## Results and Discussion

### Salinomycin inhibits cell viability and promotes anoikis

**Fig 1:**
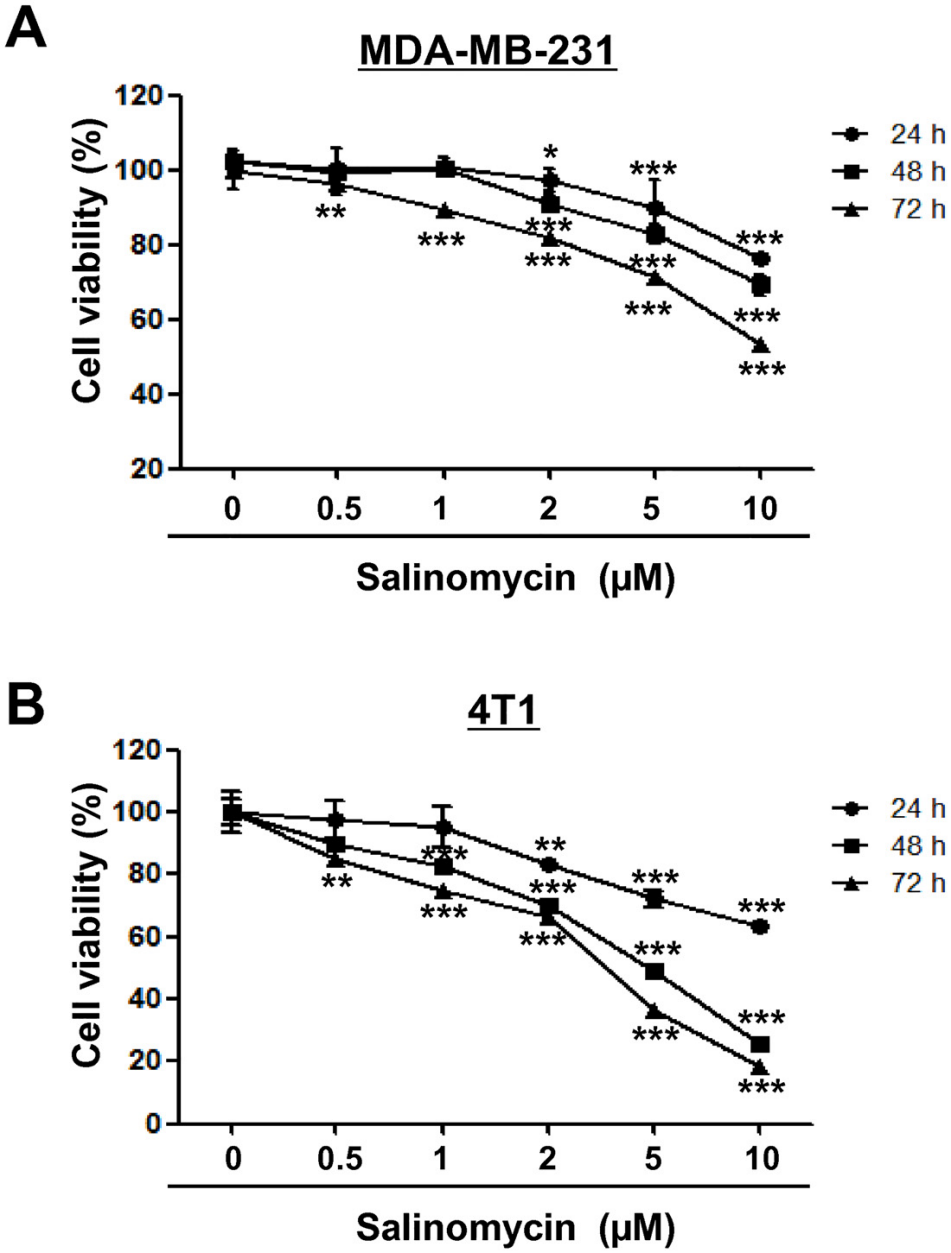
Salinomycin suppresses cell viability. TNBC cell lines [(A) MDA-MB-231 and (B) 4T1 cells] were treated with salinomycin (0.5–10 μM) or vehicle (DMSO) for the indicated durations. Viable cells were evaluated by MTS assay. Results are expressed as mean ± SEM and were analyzed by two-way ANOVA followed by Bonferroni’s *post hoc* test (*p<0.05, ** *p*<0.01 and *** *p*<0.001, versus DMSO control). The experiment was independently performed three times (n = 3). Sal, salinomycin.

**Fig 2:**
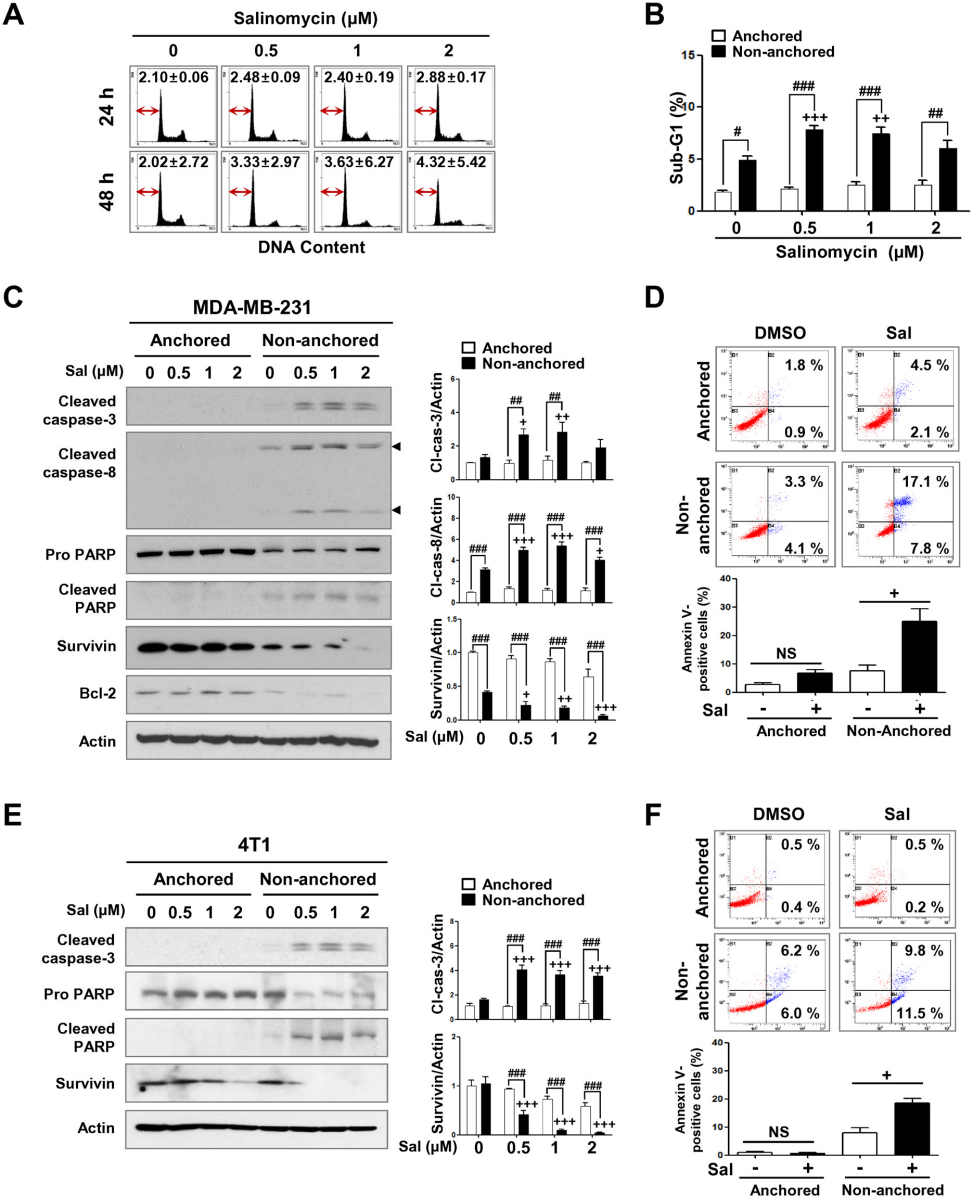
Salinomycin induces anoikis-sensitivity. (A) After exposure to salinomycin (0.5–2 μM) or DMSO for 24–48 h, the sub-G1 population was examined by flow cytometry. The percentage of cells in the sub-G1 fraction (red arrow) is depicted in the plot. (B-D) MDA-MB-231 and (E-F) 4T1 cells were grown in plates that were either uncoated or coated with poly-HEMA (2-hydroxyethyl methacrylate, 10 mg/ml) for 24 h and then treated with salinomycin (0.5–2 μM) or DMSO. (B) Effect of salinomycin on the sub-G1 fraction in anchorage-dependent and -independent conditions. The graph represents the percentage of cells in sub-G1 (++ *p*<0.01 and # *p*<0.05). (C) Effect of salinomycin on levels of apoptosis-related factors in MDA-MB-231 anchorage-dependent and -independent cells. Actin was used as a loading control. Quantitative graphs of cleaved caspase-3, cleaved caspase-8, and survivin signal intensity are shown (right panel, + *p*<0.05 and ## *p*<0.01). (D) Salinomycin (2 μM, 48 h) significantly increased the number of early and late apoptotic cells in anchorage-independent growth. The graph represents the percentage of annexin V-positive cells (bottom panel, + *p*<0.05). (E) Effect of salinomycin on levels of apoptosis-related factors in 4T1 anchorage-dependent and -independent cells. Quantification of cleaved caspase-3 and survivin are shown (right panel, +++ *p*<0.001 and ### *p*<0.001). (F) Early and late apoptotic cells were increased following salinomycin treatment (0.5 μM, 48 h) in 4T1 anchorage-independent growth. The percentage of annexin V-positive cells are shown in the graph (bottom panel, + *p*<0.05). The results are expressed as mean ± SEM and were analyzed by two-way ANOVA followed by Bonferroni’s *post hoc* test (+ *p*<0.05, ++ *p*<0.01, and +++ *p*<0.001, versus anchorage-independent DMSO control; # *p*<0.05, ## *p*<0.01, and ### *p*<0.001, versus each concentration; NS, not significant). All experiments were independently performed at least three times (n = 3).

Recent reports have demonstrated that salinomycin exerts anticancer effects by inhibiting cell growth, inducing apoptosis, and overcoming ABC transporter-mediated multidrug resistance in several cancers [29–32]. To examine the cytotoxic effect of salinomycin in MDA-MB-231 and 4T1 cells, cells were treated with salinomycin (0.5–10 μM) or DMSO as a vehicle control for the indicated durations. Salinomycin was observed to significantly decrease cell viability in a dose- and time-dependent manner (* *p*<0.05, Fig 1). Sub-G1 analysis revealed that treatment with salinomycin did not induce a statistically significant increase in sub-G1 accumulation (*p* = 0.5639, Fig 2A), with our results suggesting that 2 μM salinomycin is insufficient to trigger apoptosis.

We therefore speculated that salinomycin might be sensitizing the TNBC cells to anoikis. Cells undergo anoikis in the absence of cell-ECM interaction via intrinsic and extrinsic apoptotic pathways [16,17]. We cultured MDA-MB-231 cells in poly-HEMA coated plates to prevent cell attachment, and anoikis was then evaluated by sub-G1 analysis in the presence or absence of salinomycin (0.5–2 μM) or DMSO for 24 h. DNA content analysis revealed a significant increase in sub-G1 accumulation in anchorage-independent conditions but not in anchorage-dependent conditions (Fig 2B). Western blot analysis revealed that salinomycin-promoted anoikis accompanied by an increase in PARP cleavage and the activation of caspases 3 and 8 (Fig 2C). Cells in both early and late apoptosis were significantly increased in the presence of salinomycin in anchorage-independent conditions when compared to anchorage-dependent conditions (Fig 2D). Similar observations were seen for 4T1 cells. Salinomycin treatment (0.5–2 μM) led to PARP cleavage, activation of caspase-3 and downregulation of survivin (Fig 2E). Furthermore, salinomycin-induced anoikis-sensitivity was confirmed by increased early and late apoptotic cells in 4T1 anchorage-independent growth but not in anchorage-dependent (Fig 2F).

### Salinomycin suppresses STAT3 activation and inhibits cell migration and invasion

**Fig 3:**
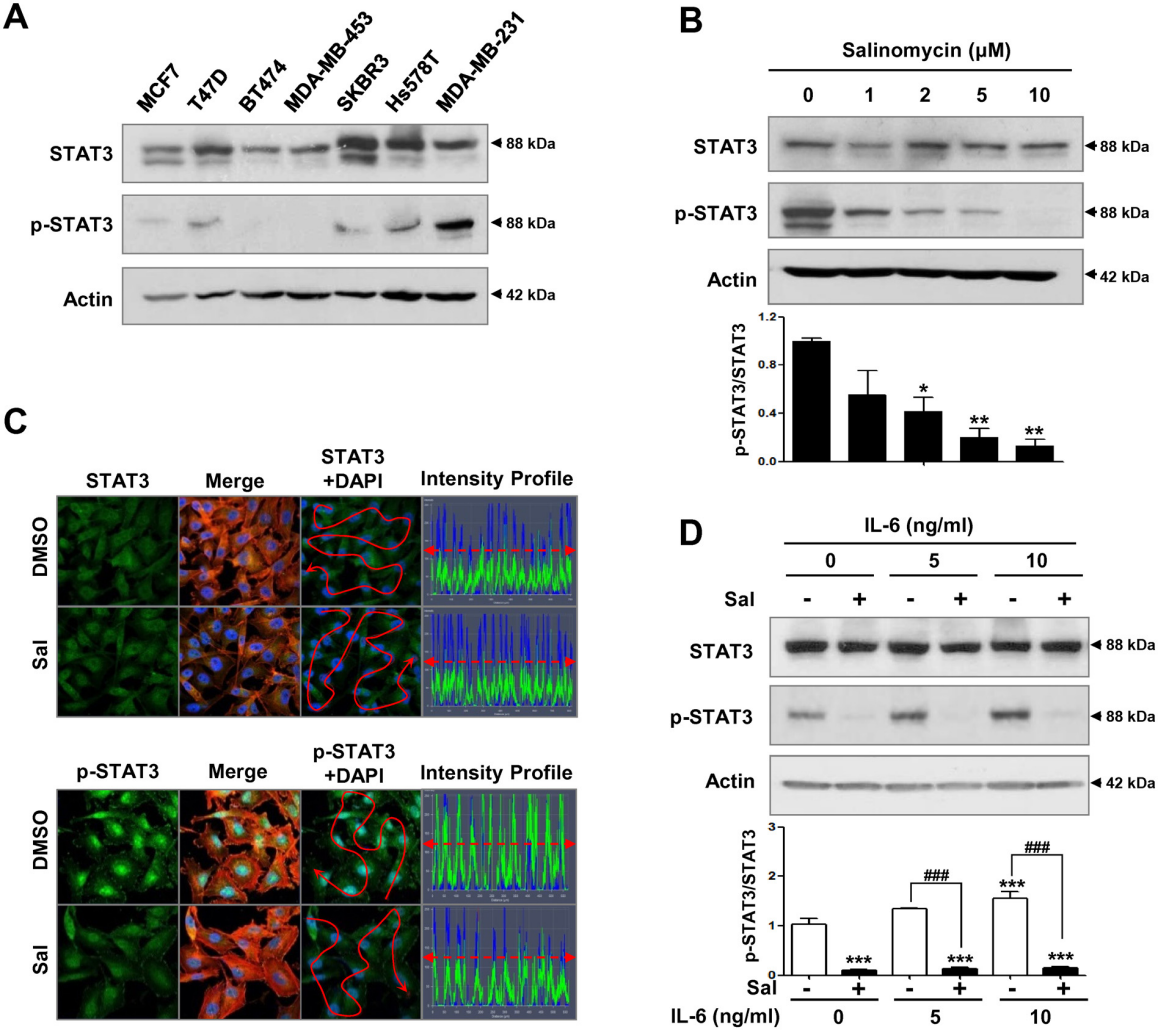
Salinomycin suppresses STAT3 activation. (A) Basal levels of STAT3 and phospho-STAT3 (Tyr705) in seven breast cancer cell lines: luminal-type (MCF7 and T47D), HER2-amplified (BT474, MDA-MB-453, and SKBR3), and TNBC (Hs578T and MDA-MB-231). (B) After exposure of MDA-MB-231 cells to salinomycin (1–10 μM) or DMSO for 48 h, STAT3 and phospho-STAT3 protein content were determined by Western blot analysis. A quantitative graph of the phospho-STAT/STAT3 ratio is shown (bottom panel, * *p*<0.05). (C) Cells were treated with salinomycin (2 μM, 48 h) and immunostained for STAT3 (1:100, green) or phospho-STAT3 (1:100, green), with DAPI nuclear staining (blue). F-actin (1:100, red) was used as a cytosolic marker. Nuclear phospho-STAT3 intensity (y-axis) is represented in arbitrary units as defined by the software. (D) Cells were pretreated with IL-6 (0–10 ng/ml) for 1 h before salinomycin treatment (2 μM, 48 h). STAT3 and phospho-STAT3 protein levels were determined by Western blot analysis. Quantification of the phospho-STAT/STAT3 ratio is shown (bottom panel, *** *p*<0.001 and ### *p*<0.001). The results are expressed as mean ± SEM (n = 3, independent experiments) and were analyzed by one-way ANOVA followed by Bonferroni’s *post hoc* test (* *p*<0.05, ** *p*<0.01 and *** *p*<0.001, versus DMSO control; ### *p*<0.001, versus each treatment).

Recent reports have shown that STAT3 activation is associated with anoikis and that phospho-STAT3 (Tyr705) levels are elevated in anoikis-resistant melanoma and pancreatic cancer cells [18,19,33]. We first compared basal levels of STAT3 and phospho-STAT3 in seven breast cancer cell lines: luminal-type (MCF7 and T47D), HER2-amplified (BT474, MDA-MB-453, and SKBR3), and TNBC (Hs578T and MDA-MB-231). STAT3 phosphorylation was comparably higher in TNBC cell lines (Fig 3A). After exposure to salinomycin (1–10 μM), phospho-STAT3 protein levels were notably reduced in a concentration-dependent manner (Fig 3B). This observation was further accompanied by decreased nuclear accumulation of phospho-STAT3, although the overall levels of STAT3 were unaffected (Fig 3C). It has been demonstrated that STAT3 is activated by the cytokine interleukin-6 (IL-6) [25,34,35]. We next examined the effect of salinomycin on STAT3 activation after IL-6 stimulation. MDA-MB-231 cells were pretreated with IL-6 (0–10 ng/ml) for 1 h, and then incubated with 2 μM salinomycin for 48 h. IL-6 significantly increased the phospho-STAT3/total-STAT3 ratio, whereas its effect was diminished by salinomycin treatment (Fig 3D). Since STAT3 activation and phosphorylation is mediated by the activation of Janus kinase 2 (JAK2) [36], we examined whether salinomycin-induced inhibition of STAT3 phosphorylation is associated with JAK2 inactivation. Although salinomycin significantly suppressed STAT3 phosphorylation (Fig 3B and S1 Fig), levels of JAK2 and phospho-JAK2 (Tyr1007/1008) were not significantly affected in MDA-MB-231 cells (S1 Fig). These results suggest that STAT3 inactivation by salinomycin is not linked with the JAK2 signaling pathway. The mechanism by which salinomycin suppresses STAT3 activation remains to be further elucidated.

**Fig 4:**
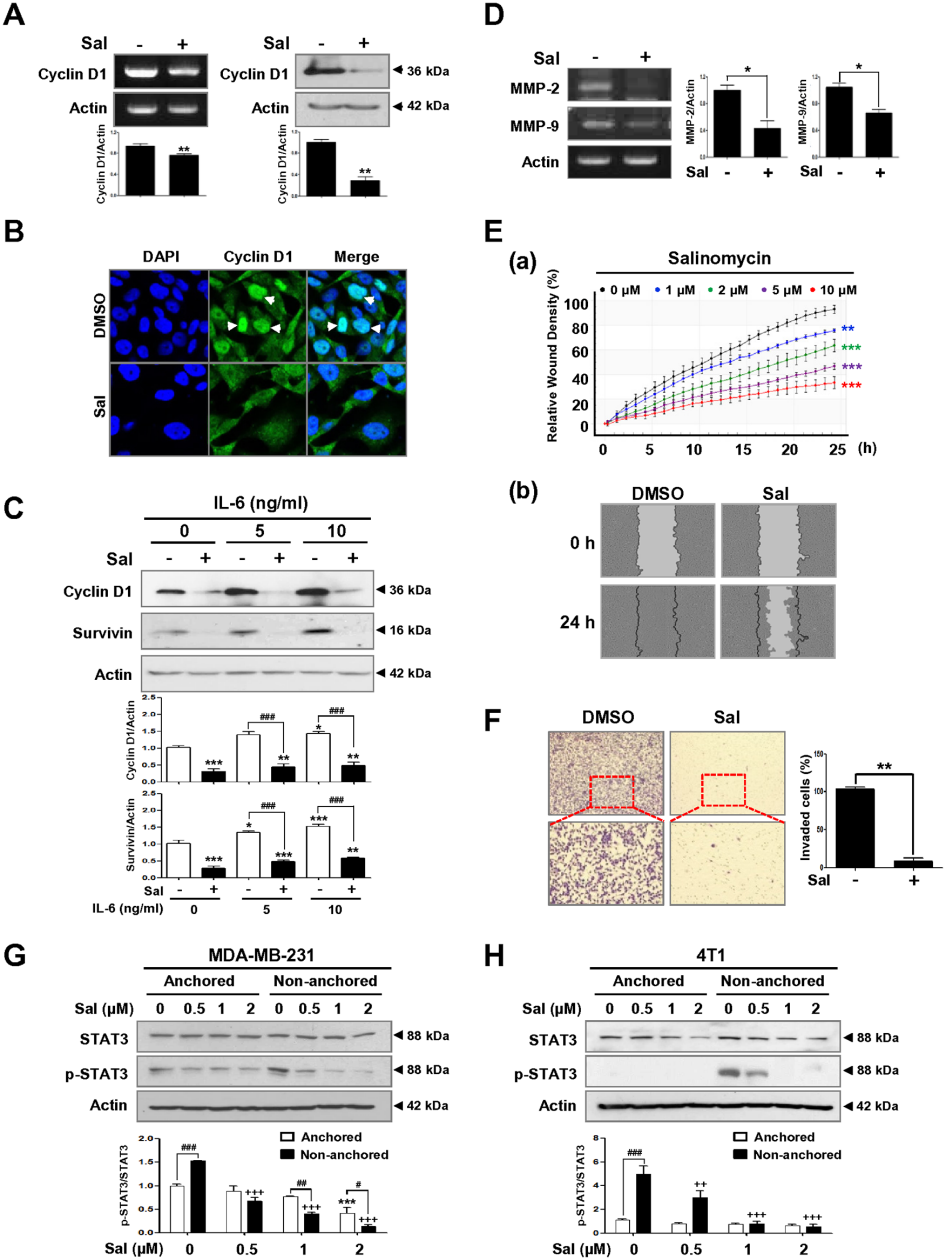
Salinomycin inhibits the expression of STAT3 downstream target molecules and impairs cell migration and invasion. (A) Effect of salinomycin (2 μM, 48 h) on cyclin D1 mRNA and protein content. Quantitative graphs of cyclin D1 mRNA and protein levels are shown (bottom panel, Student’s t-test, ** *p*<0.01). (B) Effect of salinomycin on subcellular localization of cyclin D1. Nuclear accumulation of cyclin D1 (1:100, green) was reduced following salinomycin (2 μM, 48 h) treatment. White arrow indicates nuclear cyclin D1; the nuclei were stained with DAPI. (C) Effect of salinomycin on cyclin D1 and survivin protein content in the presence or absence of IL-6 (0–10 ng/ml) in MDA-MB-231 cells. Quantitative graphs of cyclin D1 and survivin protein levels are shown (bottom panel, * *p*<0.05 and ### *p*<0.001). (D) *MMP-2* and *MMP-9* mRNA abundance was analyzed by RT-PCR analysis of total RNA isolated from DMSO- or salinomycin-treated cells (2 μM, 48 h). Quantitative graphs of *MMP-2* and *MMP-9* mRNA levels are shown (right panel, Student’s t-test, * *p*<0.05). (E) Effect of salinomycin on cell migration. After salinomycin treatment (0–10 μM), kinetic analysis of cell migration was conducted using an IncuCyte^™^ Live-Cell Imaging System for the indicated time durations. (a) The kinetic graph of cell migration represents the relative wound density (** *p*<0.01). (b) Representative images show wound closure by cell migration at 0 and 24 h in the presence or absence of 2 μM salinomycin. The black lines indicate the initial scratch areas (width, 700–800 μm) and the gray regions represent the empty space not covered by cells. (F) Effect of salinomycin on cell invasion. Images of invading cells were captured with an inverted microscope at ×200 magnification. Enlarged images from selected areas are shown. The graph represents the percentage of invaded cells (right panel, Student’s t-test, ** *p*<0.01). (G-H) Effect of salinomycin (0.5–2 μM, 24 h) on STAT3 and phospho-STAT3 protein levels in (G) MDA-MB-231 and (H) 4T1 cells in anchorage-dependent and -independent growth. Quantitative graph of intensity ratio of STAT3 and phospho-STAT3 is shown (bottom panel). The results are presented as mean ± SEM and were analyzed by two-way ANOVA followed by Bonferroni’s *post hoc* test (* *p*<0.05, ** *p*<0.01, ***p<0.001, versus anchorage-dependent DMSO control; ++ *p*<0.01 and +++ *p*<0.001, versus anchorage-independent DMSO control; # *p*<0.05, ## *p*<0.01 and ### *p*<0.001, versus each concentration). All experiments were independently performed at least three times (n = 3).

We next focused on the effect of salinomycin on STAT3 downstream target genes such as cyclin D1 and the matrix metalloproteinases MMP-2 and MMP-9 [25,37,38]. Cyclin D1 overexpression at the mRNA and protein levels is found in up to 50% of breast cancers and correlates with poor outcomes, suggesting that targeting cyclin D1 could be an effective treatment method for breast cancer [39,40]. RT-PCR and Western blot analysis revealed that salinomycin induced downregulation of cyclin D1 at both mRNA and protein levels (Fig 4A). Consistent with these observations, salinomycin treatment resulted in a marked reduction of nuclear cyclin D1 (Fig 4B). We also observed that IL-6 significantly induced upregulation of cyclin D1 and survivin, concomitant with increased STAT3 activation in MDA-MB-231 cells. However, these effects were strongly suppressed by salinomycin challenge, implying that salinomycin may act as a negative regulator of STAT3 (Fig 4C).

Similar to these findings, salinomycin has been shown to induce STAT3 inactivation and subsequently reduce expression levels of its downstream target genes such as cyclin D1, survivin, and Skp2 (S-phase kinase-associated protein 2, p27^kip1^ E3 ligase) in ovarian cancer cells *in vitro*. Furthermore, salinomycin induces destabilization of Skp2 via ubiquitin-mediated proteasomal degradation, which leads to cyclin-dependent kinase inhibitor p27^kip1^ upregulation, thereby contributing to G1 phase arrest and induction of apoptosis [41].

The MMP-9 and MMP-2 promoters contain a STAT3 binding site and are directly regulated by STAT3 activation [38]. Exposure to salinomycin significantly decreased MMP-9 and MMP-2 mRNA abundance (Fig 4D). It has been reported that MMP-9 and MMP-2 promote cell migration and invasion through the degradation of ECM components [42]. After treatment with salinomycin (1–10 μM) or DMSO, wound closure was monitored at 1-h intervals for 24 h. The kinetic analysis of cell migration revealed that salinomycin noticeably reduced migration in a time- and dose-dependent manner (Fig 4E). Significant inhibition of MDA-MB-231 migration was observed as early as 5 h following 2 μM salinomycin challenge (Fig 4E). Furthermore, a Matrigel invasion assay revealed that salinomycin treatment strongly suppressed cell invasion (Fig 4F).

Next, we examined whether salinomycin regulates STAT3 activation during the induction of anoikis. We found that basal levels of phospho-STAT3 were higher in non-anchored MDA-MB-231 cells compared to anchorage-dependent conditions (Fig 4G). Interestingly, phospho-STAT3 protein levels were minimal in the anchored 4T1 cells, but were notably elevated during anchorage-independent growth (Fig 4H). These observations suggest that STAT3 activation may be associated with anoikis resistance. Salinomycin treatment during anchorage-independent growth had a greater and dose-dependent inhibitory effect on STAT3 phosphorylation levels compared to treatment during anchorage-dependent growth (Fig 4G and 4H). However, the ratio between phospho-JAK2 and total JAK2 did not significantly change following salinomycin treatment in non-anchored MDA-MB-231 cells (S1B Fig).

### Salinomycin reduces the CD44^+^/CD24^-^ stem-like population during anchorage-independent growth

STAT3 is upregulated in CSC-like side populations (SP) [43], and inhibition of STAT3 causes the loss of stem-like characteristics concomitant with decreases in Nanog and Oct4 levels *in vitro* and reduces ovarian tumor growth *in vivo* [44].

**Fig 5:**
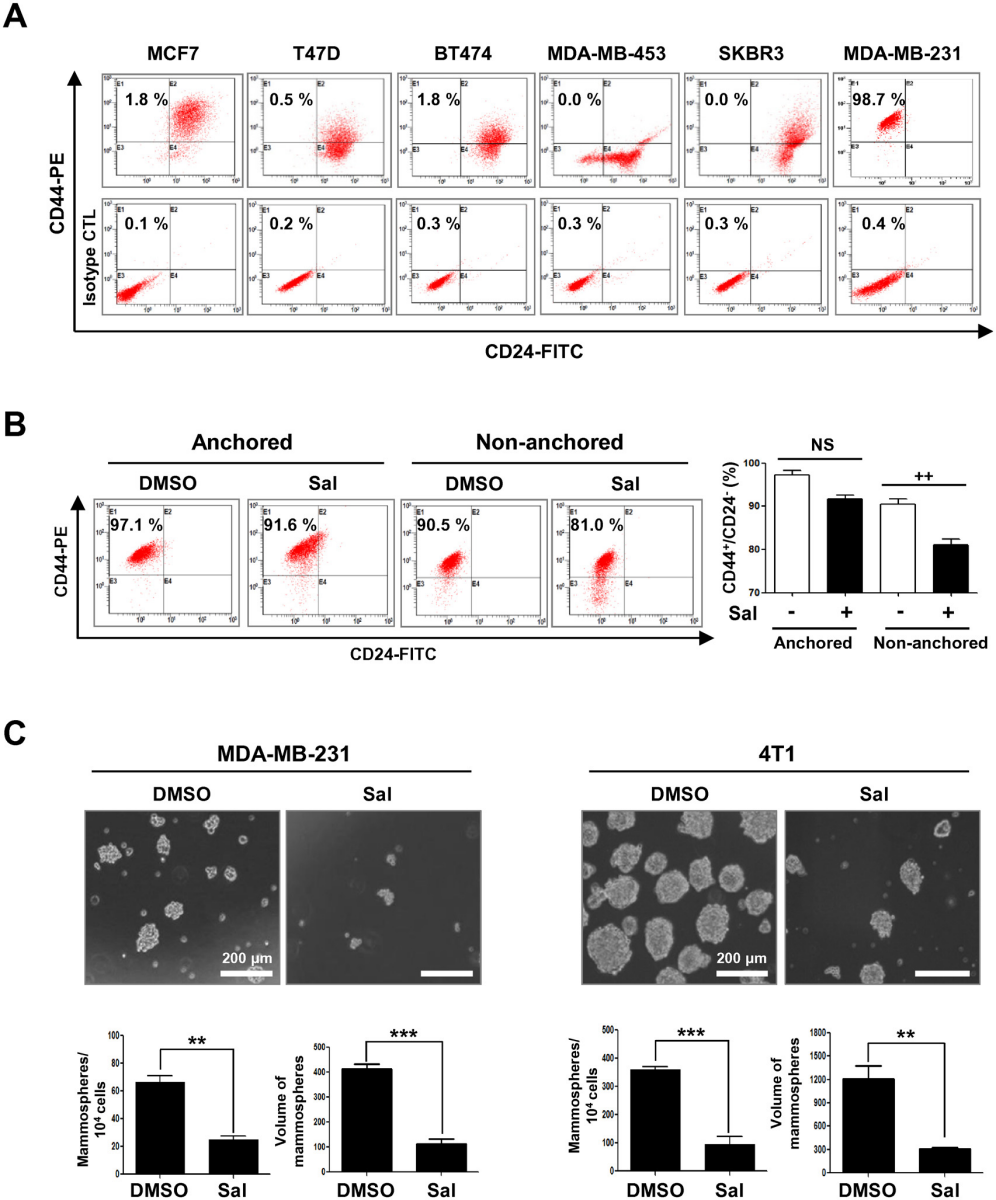
Salinomycin decreases the CD44^+^/CD24^-^ stem-like population during anchorage-independent growth and inhibits mammosphere formation. (A) The CD44^+^/CD24^-^ stem-like population in several breast cancer cell lines [luminal-type (MCF7 and T47D), HER2-amplified (BT474, MDA-MB-453, and SKBR3) and TNBC (MDA-MB-231)] was evaluated by flow cytometry. (B) Effect of salinomycin (2 μM, 48 h) on CD44^+^/CD24^-^ levels in anchorage-dependent and -independent conditions. The graph represents the percentage of CD44^+^/CD24^-^ cells (right panel). Data are expressed as mean ± SEM (n = 3, independent experiments) and were analyzed by two-way ANOVA followed by Bonferroni’s *post hoc* test (++ *p*<0.01, versus anchorage-independent DMSO control; NS, not significant). (C) MDA-MB-231 and 4T1 mammospheres were cultured for 5 days in serum-free suspension conditions in the presence or absence of salinomycin (2 μM). Graphs represents the number (per 10^4^ cells) and volumes (mm^3^) of mammospheres (bottom panel, Student’s t-test, ** *p*<0.01).

To investigate the inherent CSC-like phenotype, we first screened for CD44^+^/CD24^-^ levels in several breast cancer cell lines. The results revealed that MDA-MB-231 cells harbored the highest CD44^+^/CD24^-^ stem-like population, at approximately 98% (Fig 5A). To examine the effect of salinomycin on the CSC-like population, CD44^+^/CD24^-^ levels were evaluated in anchorage-dependent and -independent states after salinomycin treatment for 48 h. Salinomycin induced a slight, but not significant, decrease in the CD44^+^/CD24^-^ stem-like population in MDA-MB-231 anchorage-dependent growth, whereas non-anchored cells exhibited a significant decrease in CD44^+^/CD24^-^ levels (Fig 5B).

Mammosphere assays can be useful for investigating breast cancer stem cell-like behavior, as they typically contain high numbers of undifferentiated progenitor cells [13]. To investigate whether salinomycin impairs mammosphere forming ability in TNBC cell lines, MDA-MB-231 and 4T1 cells were cultured as mammospheres for 5 days in serum-free suspension conditions in the presence or absence of salinomycin (2 μM). The result revealed that salinomycin greatly inhibited the number and volume of mammospheres derived from both cell lines (Fig 5C).

Aldehyde dehydrogenase 1 (ALDH1) has emerged as a CSC marker in breast cancer [10,45], prompting us to examine the effect of salinomycin on ALDH1 activity. ALDEFLUOR positivity assays revealed that ALDH1 activity was significantly suppressed in response to salinomycin during anchorage-independent growth of MDA-MB-231 cells (S2A Fig). A recent clinical study with a breast cancer tissue microarray showed that the ALDH1 levels are significantly correlated with nuclear expression of phospho-STAT3 (Tyr705). In addition, breast cancer cells with an ALDH^+^ subpopulation exhibit a relatively higher level of phospho-STAT3 compared with ALDH^-^ cells *in vitro* [46]. Therefore, it is conceivable that suppression of STAT3 activity by salinomycin may lead to inhibition of ALDH1 activity. Defining the interplay between STAT3 activation and ALDH1 activity could provide important clues for understanding the action of CSCs in TNBC. The transcription factors Nanog and Sox2 have been identified as embryonic stem cell (ESC) markers that maintain pluripotency [47]. Although it is known that Nanog and Sox2 control tumorigenesis, salinomycin did not affect *Nanog* and *Sox2* mRNA expression in non-anchored MDA-MB-231 cells (S2B Fig).

### Combination treatment with STAT3 inhibitors and salinomycin induces apoptosis and reduces the CD44^+^/CD24^-^ stem-like population

**Fig 6:**
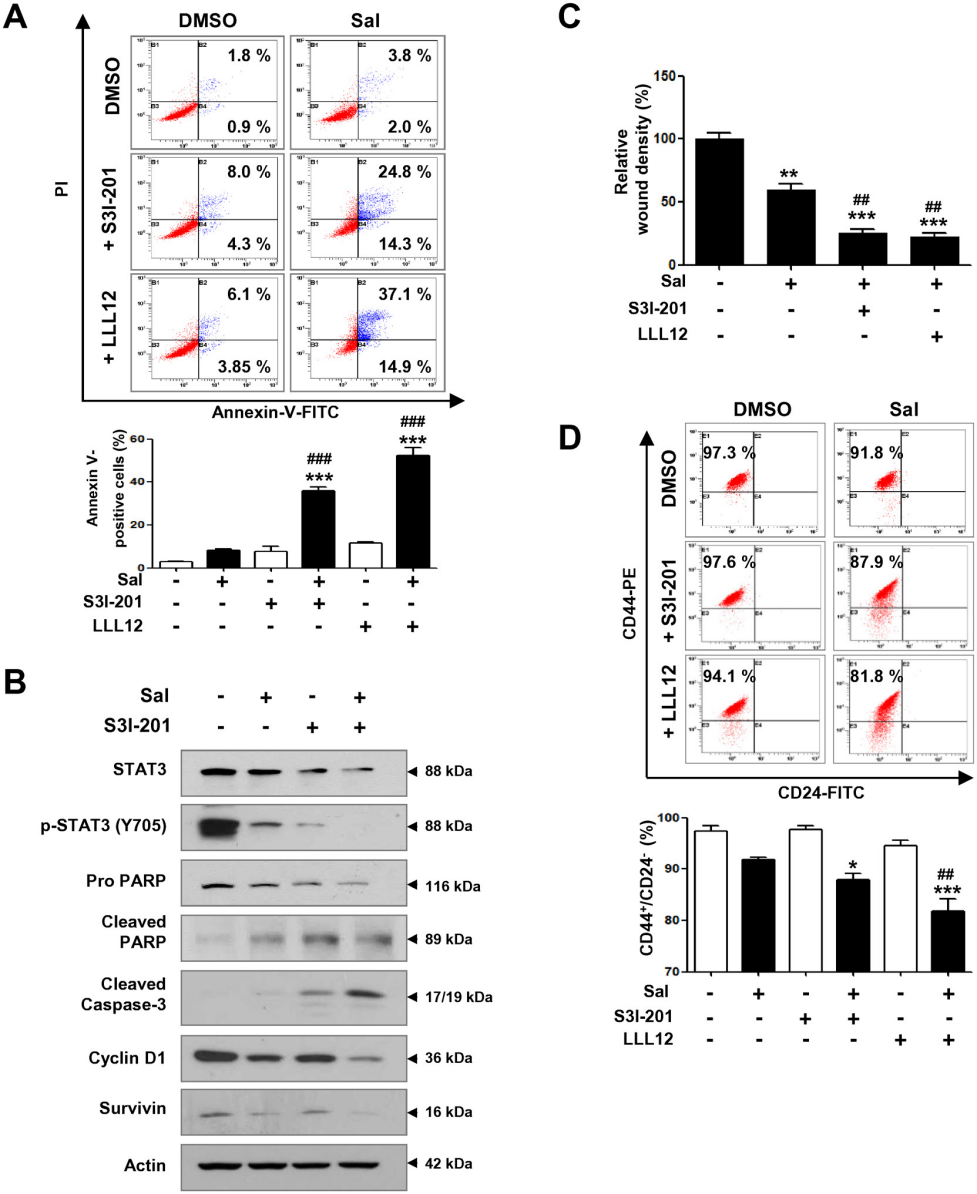
Combination effect of salinomycin and STAT3 inhibitors on apoptosis, migration, and CD44^+^/CD24^-^ stem-like population. (A) Cells were treated with salinomycin (2 μM, 48 h) and/or S3I-201 (50 μM) or LLL12 (1 μM) and annexin V/PI analysis was performed. Quantitative graph of annexin V-positive population is shown (bottom panel, *** *p*<0.001 and ### *p*<0.001). (B) Effect of combined treatment with salinomycin and S3I-201 on protein expression of STAT3, phospho-STAT3, cyclin D1, and apoptosis-related factors. (C) Cells were co-treated with salinomycin (2 μM) and S3I-201 (50 μM) or LLL12 (1 μM) and the relative wound density was assessed over 24 h. Quantitative graph of relative migration is presented (** *p*<0.01 and ## *p*<0.01). (D) Effect of salinomycin (2 μM, 48 h) combined with S3I-201 (50 μM) or LLL12 (1 μM) on the CD44^+^/CD24^-^ stem-like population. The graph represents the percentage of CD44^+^/CD24^-^ cells (bottom panel, * *p*<0.05 and ## *p*<0.01). The results were expressed as mean ± SEM and analyzed by one-way ANOVA followed by Bonferroni’s *post hoc* test (* *p*<0.05, ** *p*<0.01, and *** *p*<0.001, versus DMSO control; ## *p*<0.01 and ### *p*<0.001, versus salinomycin only). All experiments were independently performed at least three times (n = 3).

Finally, we applied combination treatment with STAT3 inhibitors and salinomycin to enhance the induction of apoptosis and suppression of the CSC population during anchorage-dependent growth. For this study, we selected two pharmacological inhibitors of STAT3 phosphorylation, S3I-201 and LLL12 [26,48]. The number of early and late apoptotic cells was significantly increased by combination treatment but not by salinomycin, S3I-201, or LLL12 alone (Fig 6A). This observation was supported by Western blot analysis showing enhanced PARP cleavage, activation of caspase-3, and a decrease in cyclin D1 protein content after combination treatment with salinomycin and S3I-201 (Fig 6B). Co-administration of salinomycin and S3I-201 also remarkably suppressed phospho-STAT3 protein levels. To further examine the effect of salinomycin in the absence or presence of STAT3 inhibitors on cell migration, we performed a wound healing assay. Cells were co-treated with salinomycin (2 μM) and S3I-201 (50 μM) or LLL12 (1 μM) for 24 h. Co-administration resulted in greatly reduced cell migration (Fig 6C). Furthermore, combination treatment resulted in a significant decrease in the CD44^+^/CD24^-^ stem-like population, whereas neither salinomycin nor STAT3 inhibitors alone could induce this effect (Fig 6D).

In summary, our observations have shown that salinomycin sensitizes TNBC cells to anchorage-independent cell death, and decreases the mammosphere formation and CD44^+^/CD24^-^ stem-like population during anchorage-independent growth. These responses were associated with the inhibition of STAT3 activation. Salinomycin also impaired cell migration and invasion in MDA-MB-231 cells. These results suggest that salinomycin may have potential for development as a novel treatment for TNBC. Further studies using *in vivo* models are needed to support these observations.

## Supporting Information

S1 Fig Salinomycin does not affect JAK2 activation. (A) After exposure to salinomycin (1–5 μM) or DMSO for 48 h, protein levels of JAK2, phospho-JAK2 (Tyr1007/1008), STAT3, and phospho-STAT3 (Tyr705) were determined using Western blot analysis. Quantitative graphs of phospho-JAK2/JAK2 ratio and phospho-STAT/STAT3 ratio are shown (bottom panel, *** *p*<0.001). (B) Effect of salinomycin (2 μM, 48 h) on activation of JAK2 and STAT3 in anchorage-dependent and -independent cells. The graphs represent the ratios of phospho-JAK2/JAK2 and phospho-STAT3/STAT3 (bottom panel, *** *p*<0.001, versus anchorage-dependent DMSO control; +++ *p*<0.001, versus anchorage-independent DMSO control). The results are presented as mean ± SEM (n = 3 independent experiments) and were analyzed by one- or two-way ANOVA as appropriate, followed by Bonferroni’s *post hoc* test.
(PDF)

S2 Fig Salinomycin reduces ALDH1 activity. (A-B) MDA-MB-231 cells were treated with salinomycin (2 μM) or DMSO for 48 h in anchorage-independent conditions. (A) Effect of salinomycin on ALDH1 activity. Cells were incubated with the ALDH protein substrate BAAA in the presence or absence of DEAB (a specific inhibitor of ALDH) to define the ALDEFLUOR-positive cell population. Quantification of ALDEFLUOR-positive population (right panel, Student’s t-test, *** *p*<0.001). (B) Effect of salinomycin on mRNA expression of *Nanog* and *Sox2*. Quantitative graphs represent mRNA abundance of *Nanog* and *Sox2* (right panel, Student’s t-test; NS, not significant). The *p* values are displayed. All experiments were independently repeated 3 times (n = 3).
(PDF)

S1 File Supplemental Materials and Methods for S2 Fig. (DOCX)

S1 Table List of reagents used in this study. (PDF)

S2 Table List of antibodies used in this study. (PDF)
