# Supplementary figures and images for: Salinomycin Promotes Anoikis and Decreases the CD44+/CD24- Stem-Like Population via Inhibition of STAT3 Activation in MDA-MB-231 Cells
Source: PLoS One. 2015 Nov 3;10(11):e0141919. doi: 10.1371/journal.pone.0141919 (PMC4631341; doi:10.1371/journal.pone.0141919)

## S1 Fig

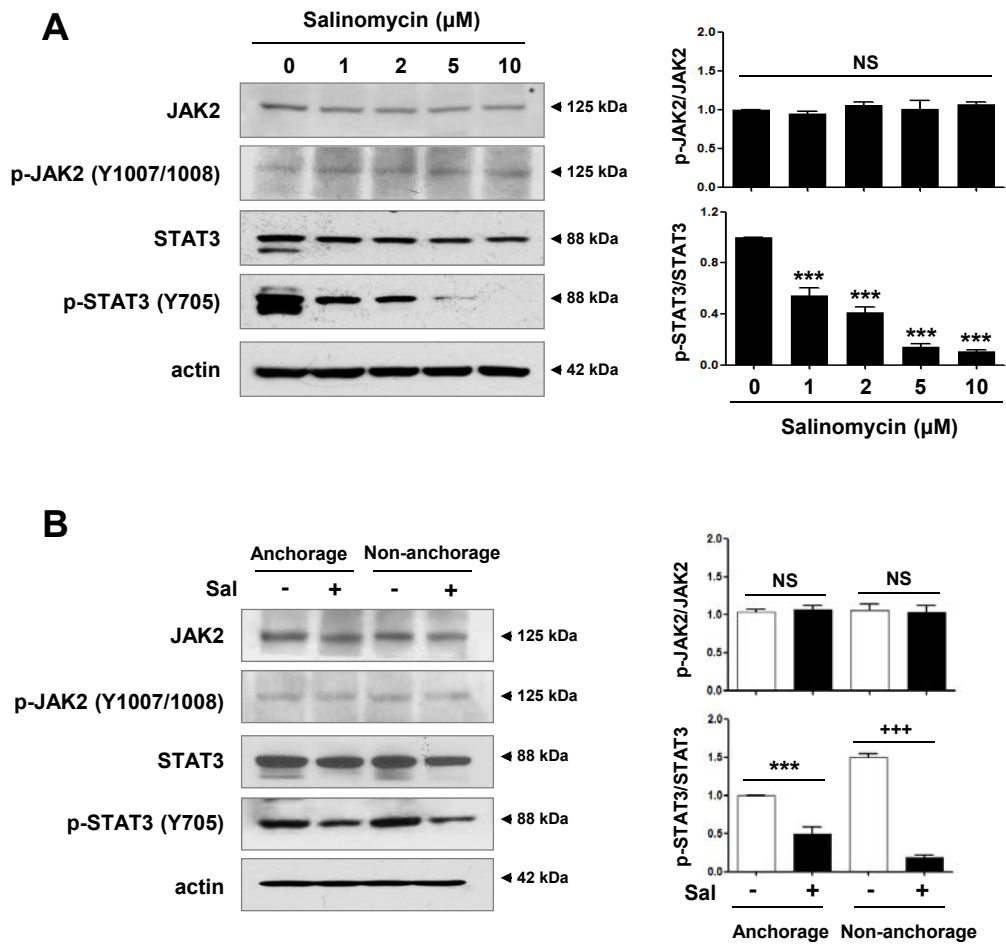

Supplement: S1 Fig — (A) After exposure to salinomycin (1–5 μM) or DMSO for 48 h, protein levels of JAK2, phospho-JAK2 (Tyr1007/1008), STAT3, and phospho-STAT3 (Tyr705) were determined using Western blot analysis. Quantitative graphs of phospho-JAK2/JAK2 ratio and phospho-STAT/STAT3 ratio are shown (bottom panel, *** p<0.001). (B) Effect of salinomycin (2 μM, 48 h) on activation of JAK2 and STAT3 in anchorage-dependent and -independent cells. The graphs represent the ratios of phospho-JAK2/JAK2 and phospho-STAT3/STAT3 (bottom panel, *** p<0.001, versus anchorage-dependent DMSO control; +++ p<0.001, versus anchorage-independent DMSO control). The results are presented as mean ± SEM (n = 3 independent experiments) and were analyzed by one- or two-way ANOVA as appropriate, followed by Bonferroni’s post hoc test. (PDF) [file pone.0141919.s001.pdf]

S2 Fig

A

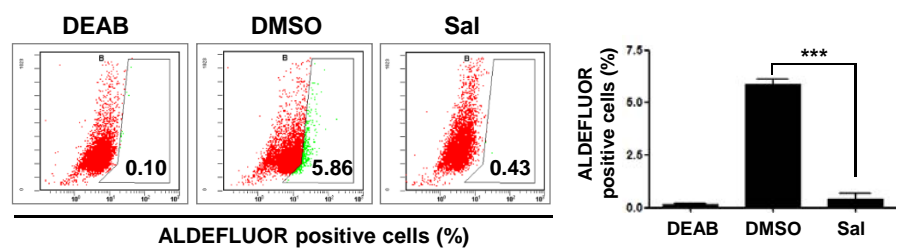

B

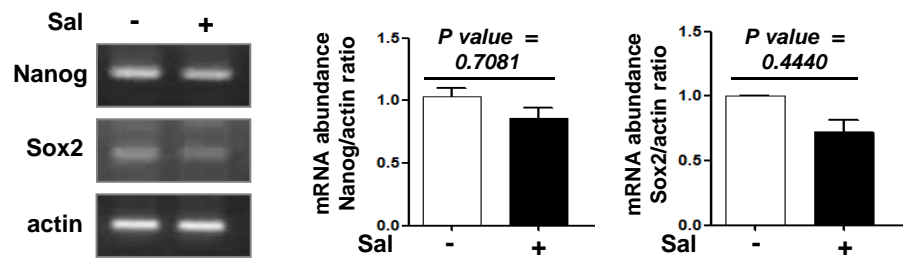

Supplement: S2 Fig — (A-B) MDA-MB-231 cells were treated with salinomycin (2 μM) or DMSO for 48 h in anchorage-independent conditions. (A) Effect of salinomycin on ALDH1 activity. Cells were incubated with the ALDH protein substrate BAAA in the presence or absence of DEAB (a specific inhibitor of ALDH) to define the ALDEFLUOR-positive cell population. Quantification of ALDEFLUOR-positive population (right panel, Student’s t-test, *** p<0.001). (B) Effect of salinomycin on mRNA expression of Nanog and Sox2. Quantitative graphs represent mRNA abundance of Nanog and Sox2 (right panel, Student’s t-test; NS, not significant). The p values are displayed. All experiments were independently repeated 3 times (n = 3). (PDF) [file pone.0141919.s002.pdf]
