# Supplementary material for: Salinomycin Promotes Anoikis and Decreases the CD44+/CD24- Stem-Like Population via Inhibition of STAT3 Activation in MDA-MB-231 Cells
Source: PLoS One. 2015 Nov 3;10(11):e0141919. doi: 10.1371/journal.pone.0141919 (PMC4631341; doi:10.1371/journal.pone.0141919)
Supplement: S1 File — (DOCX) [file pone.0141919.s003.docx]

**S1 File. Supplemental Materials and Methods for S2 Fig.**

**ALDEFLUOR assay**

The ALDEFLUOR assay kit (Stemcell Technology, Vancouver, BC) was used to assess aldehyde dehydrogenase (ALDH) activity according to the manufacturer’s protocol. Cells were incubated for 45 min at 37^o^C in Aldefluor assay buffer containing the ALDH protein substrate BODIPY-aminoacetaldehyde (BAAA, 1 µM per 0.5 ⨯ 10⁶ cells). As a specific inhibitor of ALDH1, 50 mM diethylamino-benzaldehyde (DEAB) was used for defining the Aldefluor population. Aldefluor stained cells were analyzed with a Beckman Coulter Expo flow cytometer (Beckman Coulter, Brea, CA).

**RT-PCR analysis**

Total RNA was extracted using the RNeasy mini kit (Qiagen, Valencia, CA, USA), according to the manufacturer’s instructions. Amplification of transcripts was performed by reverse transcriptase-polymerase chain reaction (RT-PCR) using 1 μg/μl total RNA, Molony Murine Leukemia Virus reverse transcriptase (MMLV; Gibco/BRL, Gaithersburg, MD, USA), and oligo-d(T)15 primer (Roche Applied Sciences). PCR amplification was performed using the following primers: Nanog, forward 5’-TTG TGC GCC TGA AGA AAA CTA TCC-3’, reverse 5’-CTG CGT CAC ACC ATT GCT ATT CTT-3’; Oct4, forward 5’-GAC AAC AAT GAG AAC CTT CAG GAG A-3’, reverse : 5’-CTG GCG CCG GTT ACA GAA CCA-3’; Sox2, forward 5’-CCC CCG GCG GCA ATA GCA-3’, reverse : 5’-TCG GCG CCG GGG AGA TAC AT-3’; actin, forward 5’-ACC CAG ATC ATG TTT GAG AC-3’, reverse 5’-GGA GTT GAA GGT AGT TTC GT-3’. The PCR products were separated on 1.2 % agarose gels and visualized using a Gel Doc™ XR+ System (Bio-Rad Laboratories).
