# Supplementary material for: Salinomycin Promotes Anoikis and Decreases the CD44+/CD24- Stem-Like Population via Inhibition of STAT3 Activation in MDA-MB-231 Cells
Source: PLoS One. 2015 Nov 3;10(11):e0141919. doi: 10.1371/journal.pone.0141919 (PMC4631341; doi:10.1371/journal.pone.0141919)
Supplement: S1 Table — (PDF) [file pone.0141919.s004.pdf]

S1 Table. List of reagents used in this study

Reagents	Manufacturer, catalog #	Diluted solution
FBS (fetal bovine serum)	Gibco (Cat #16000-044)	
DMSO (dimethyl sulfoxide)	SIGMA (Cat # D2650)	
Triton X-100	SIGMA (Cat # X100)	
Tween-20	Amresco (Cat # 0777)	
PFA (paraformaldehyde)	SIGMA (Cat # P6148)	
PBS (phosphate buffered saline) tablet	SIGMA (Cat # P4417)	
phosphatase inhibitor cocktail	Roche (Cat # 04906845001)	
protease inhibitor cocktail	Roche (Cat # 11836153001)	
Salinomycin	SIGMA (Cat # S6201)	DMSO
S3I-201	SIGMA (Cat # SML-0330)	DMSO
LLL12	Biovision Inc. (Cat # 1792-5)	DMSO
FITC Annexin V Apoptosis Detection kit 1	BD biosciences (Cat # 555647)	
CellTiter 96* Aqueous One Solution Cell Proliferation Assay	Promega (Cat # G3580)	
ProLong Gold Antifade Reagent with DAPI	Life Technologies (Cat # P36931)	
antibody diluent	Dako (Cat # S0809)	
Matrigel matrix	BD biosciences (Cat # 354230)	
RNeasy mini kit	Quiagen (Cat # 74106)	
PI (propidium iodide) solution	SIGMA (Cat # P4864)	
RNase	Life Technologies (Cat # 12091)	
ALDEFLUOR™ Kit	Stem cell Technologies (Cat # 01700)	
PE Anti-Human CD44	BD biosciences (Cat # 555479)	
FITC Anti-human CD24	BD biosciences (Cat # 555427)	
Interleukin-6 (IL-6)	SIGMA (Cat # SRP3096)	
