# Supplementary material for: Salinomycin Promotes Anoikis and Decreases the CD44+/CD24- Stem-Like Population via Inhibition of STAT3 Activation in MDA-MB-231 Cells
Source: PLoS One. 2015 Nov 3;10(11):e0141919. doi: 10.1371/journal.pone.0141919 (PMC4631341; doi:10.1371/journal.pone.0141919)
Supplement: S2 Table — (PDF) [file pone.0141919.s005.pdf]

S2 Table. List of antibodies used in this study

Name of Antibody	Manufacturer, catalog #	Species raised in;	Dilution used
Cleaved Caspase-3 (Asp175)	Cell Signaling (Cat # 9664)	Rabbit Monoclonal	1:1000 (WB)
Cleaved Caspase-8 (Asp391)	Cell Signaling (Cat # 9496)	Rabbit Monoclonal	1:1000 (WB)
PARP	Cell Signaling (Cat # 9542)	Rabbit Polyclonal	1:2000 (WB)
Cleaved PARP (Asp214)	Cell Signaling (Cat #5625)	Rabbit Monoclonal	1:2000 (WB)
survivin (FL-142)	Santa Cruz (Cat # sc-10811)	Rabbit Polyclonal	1:2000 (WB)
Bcl-2 Clone 124	DAKO (Cat # M0887)	Mouse Monoclonal	1:1000 (WB)
JAK2 (D2E12)	Cell Signaling (Cat # 3230)	Rabbit Monoclonal	1:2000 (WB)
Phospho-JAK2 (Tyr1007/1008) (C80C3)	Cell Signaling (Cat # 3776)	Rabbit Monoclonal	1:2000 (WB)
STAT3	abcam (Cat # ab50761)	Mouse Monoclonal	1:100 (IF), 1:2000 (WB)
Anti-STAT3 (phospho Y705)	abcam (Cat # ab76315)	Rabbit Monoclonal	1:100 (IF), 1:2000 (WB)
cyclin D1 (DCS-6)	Santa Cruz (Cat # sc-20044)	Mouse Monoclonal	1:100 (IF), 1:2000 (WB)
β-actin	SIGMA (Cat # A5316)	Mouse Monoclonal	1:5000 (WB)
Rhodamine Phalloidin	Invitrogen (Cat # R415)		1:100 (IF)
HRP-conjugated anti-rabbit	Bio-rad (Cat # 170-6515)		1:3000-10000 (WB)
HRP-conjugated anti-mouse	Bio-rad (Cat # 172-1011)		1:3000-10000 (WB)
Alexa Fluor-488 goat anti-mouse IgG	Invitrogen (Cat # A11001)	Mouse polyclonal	1:100 (IF)
Alexa Fluor-488 goat anti-rabbit IgG	Invitrogen (Cat # A11008)	Rabbit Polyclonal	1:100 (IF)
Western Blot (WB), Immunofluorescence (IF)			
